# Quantitative dual-tracer PET/CT biomarkers correlate concordant lesion uptake with PSMA-RLT outcomes in mCRPC: a dual-center study

**DOI:** 10.1007/s00259-025-07700-6

**Published:** 2026-01-05

**Authors:** Song Xue, Holger Einspieler, Sijie Wen, Dina Muin, Ana Antic Nikolic, Jan Baessler, Gero Kramer, Shahrokh F. Shariat, Constantin Lapa, Marcus Hacker, Sazan Rasul, Xiang Li

**Affiliations:** 1 Division of Nuclear Medicine, Department of Biomedical Imaging and Image-Guided Therapy, Vienna General Hospital, Medical University of Vienna, 1090 Vienna, Austria; 2 Department of Urology, Medical University of Vienna, Vienna, Austria; 3 Department of Urology, Weill Cornell Medical College, New York, NY USA; 4 Department of Urology, University of Texas Southwestern, Dallas, TX USA; 5 Karl Landsteiner Institute of Urology and Andrology, Vienna, Austria; 6 Department of Nuclear Medicine, Beijing Chest Hospital, Capital Medical University, Beijing, China; 7 Nuclear Medicine, Faculty of Medicine, University of Augsburg, Augsburg, Germany

**Keywords:** PSMA-RLT, PSMA PET/CT, FDG PET/CT, Fusion PET, Prognosis

## Abstract

**Abstract:**

Prostate-specific membrane antigen radioligand therapy (PSMA-RLT) has emerged as a promising treatment for metastatic castration-resistant prostate cancer (mCRPC). However, current patient selection methods – largely based on qualitative imaging criteria – may impede precision and efficacy of treatment. We aimed to evaluate the predictive value of quantitative imaging biomarkers derived from dual-tracer [^68^ Ga]Ga-PSMA-11 and [^18^F]F-FDG PET/CT, with a focus on concordant lesions.

**Methods:**

Thirty-seven mCRPC patients from two institutions underwent [^68^ Ga]Ga-PSMA-11 and [^18^F]F-FDG PET/CT prior to receiving at least two cycles of [^177^Lu]Lu-PSMA therapy. An automated pipeline enabled lesion segmentation, dual-tracer image fusion, and extraction of quantitative features from concordant (PSMA + /FDG +) and non-concordant lesions. A decision tree model was developed on the Vienna cohort (*n* = 24) and validated on an independent cohort from Augsburg (*n* = 13). SHAP analysis was used to identify key predictive features.

**Results:**

The decision tree achieved 95.8% accuracy in the training cohort and 84.6% in external validation. SUV_mean_ of concordant lesions was the most predictive features. Patients with SUV_mean_[PSMA Concordant] ≥ 12.1 g/mL were more likely to respond. Organ-specific analysis further identified high SUV_max_ in bone metastases as a negative prognostic marker.

**Conclusions:**

Quantitative metrics from dual-tracer PET, particularly those characterizing concordant lesions, show promise for predicting response to PSMA-RLT. These preliminary findings highlight the potential to move beyond binary eligibility criteria toward a more nuanced, biomarker-driven approach to patient selection.

## Introduction

Prostate-specific membrane antigen radioligand therapy (PSMA-RLT) has emerged as a promising therapeutic option for patients with metastatic castration-resistant prostate cancer (mCRPC) [1]. However, clinical outcomes remain heterogeneous, with a substantial proportion of patients failing to respond—even when selected based on PSMA-positivity on PET imaging [2]. In the pivotal VISION phase 3 trial [3], all enrolled patients demonstrated PSMA expression on PET, yet only 46% achieved a ≥ 50% decline in prostate-specific antigen (PSA) following treatment with [^177^Lu]Lu-PSMA-617. This variability underscores the limitations of current imaging-based selection criteria, and highlights the need for more refined selection biomarkers beyond the presence of PSMA uptake alone.

A promising approach to improve patient stratification is dual-tracer PET imaging using both a PSMA-targeted radiotracer and [^18^F]F-FDG [4, 5]. While PSMA PET visualizes target expression, FDG PET provides complementary information on tumor metabolic activity, particularly in aggressive or dedifferentiated lesions. In current practice, dual-tracer imaging is often interpreted in a binary fashion – for instance, declaring a patient ineligible for PSMA-RLT if any “mismatch” lesion is present (PSMA-negative and FDG-positive). While this binary yes/no assessment of PSMA–FDG concordance helps exclude certain non-ideal candidates, it fails to leverage the full quantitative potential of dual PET imaging. The TheraP biomarker analysis demonstrated that continuous PSMA SUV_mean_ and FDG disease burden provide independent, quantitative prognostic/predictive information—beyond binary mismatch rules [6]. Although such patient-level quantitative metrics are increasingly reported, few frameworks operate at the lesion-subset level to integrate PSMA intensity and FDG activity within PSMA/FDG-concordant disease.

We hypothesized that a more quantitative analysis of dual-tracer PET, with a particular focus on concordant lesions, could yield reliable imaging biomarkers predictive of therapeutic response. To achieve this, we developed an automated image fusion and lesion segmentation pipeline for co-registered [^68^ Ga]Ga-PSMA-11 and [^18^F]F-FDG PET/CT. In contrast to analysis such as TheraP, which analyzed whole-body PSMA and FDG features independently at the patient level, our approach specifically quantifies concordant lesions, defined as regions simultaneously PSMA- and FDG-avid, thereby capturing intrapatient heterogeneity beyond global averages. Using the extracted imaging features, we applied a machine learning–based decision tree model to evaluate their predictive value for PSA response following PSMA-RLT. Our goal is to enhance patient selection for PSMA-RLT by moving beyond simplistic concordance criteria towards a multifactorial imaging biomarker approach that is both quantitative and clinically actionable.

## Materials and methods

### Patient population

Patients with mCRPC who underwent dual-tracer PET/CT imaging prior to the PSMA-RLT were included from two centers (Medical University of Vienna and University of Augsburg), following an identical imaging and treatment protocol. All patients received at least two cycles of [^177^Lu]Lu-PSMA therapy after imaging. Written informed consent was obtained from all participants, and the study was approved by the institutional review board in accordance with the Declaration of Helsinki, and approved by the Vienna General Hospital, Medical University of Vienna Institutional Review Board (Ethic No:1745/2021). All patients provided written informed consent prior to participation.

### Outcome

Therapeutic response was assessed by the relative change in serum PSA levels from baseline (pre-RLT) to post–cycle 2 of PSMA-RLT, patients were categorized into three response groups [3]: Progressive Disease (PD): Any PSA increase from baseline; Partial Response (PR): PSA decline < 50% from baseline; Complete Response (CR): PSA decline ≥ 50% from baseline. These group definitions were used as class labels for subsequent predictive modeling.

### Imaging protocols

All patients routinely underwent PET/CT imaging with [^68^ Ga]Ga-PSMA-11 first, followed by [^18^F]F-FDG within a two-week interval. Whole body scans (skull to mid-thigh) were performed on a modern hybrid PET/CT scanner and were obtained 45–60 min after the administration. For PSMA, diagnostic CT scans were usually conducted using 80–140 kV and 80–180 mAs. For FDG, low-dose CT scans were usually performed using 80–120 kV and 10–40 mAs to limit cumulative radiation exposure. A slice thickness of 2 mm and a matrix of 512 × 512 was used in both scans. PET images were reconstructed with iterative algorithms and standard corrections for attenuation, scatter, and decay. Depending on the acquisition protocol, PET images were reconstructed with a matrix size of either 440 × 440 or 220 × 220. All quantitative PET uptake values were reported in units of standardized uptake value (SUV), normalized to body weight.

### Imaging feature analysis

**Fig. 1:**
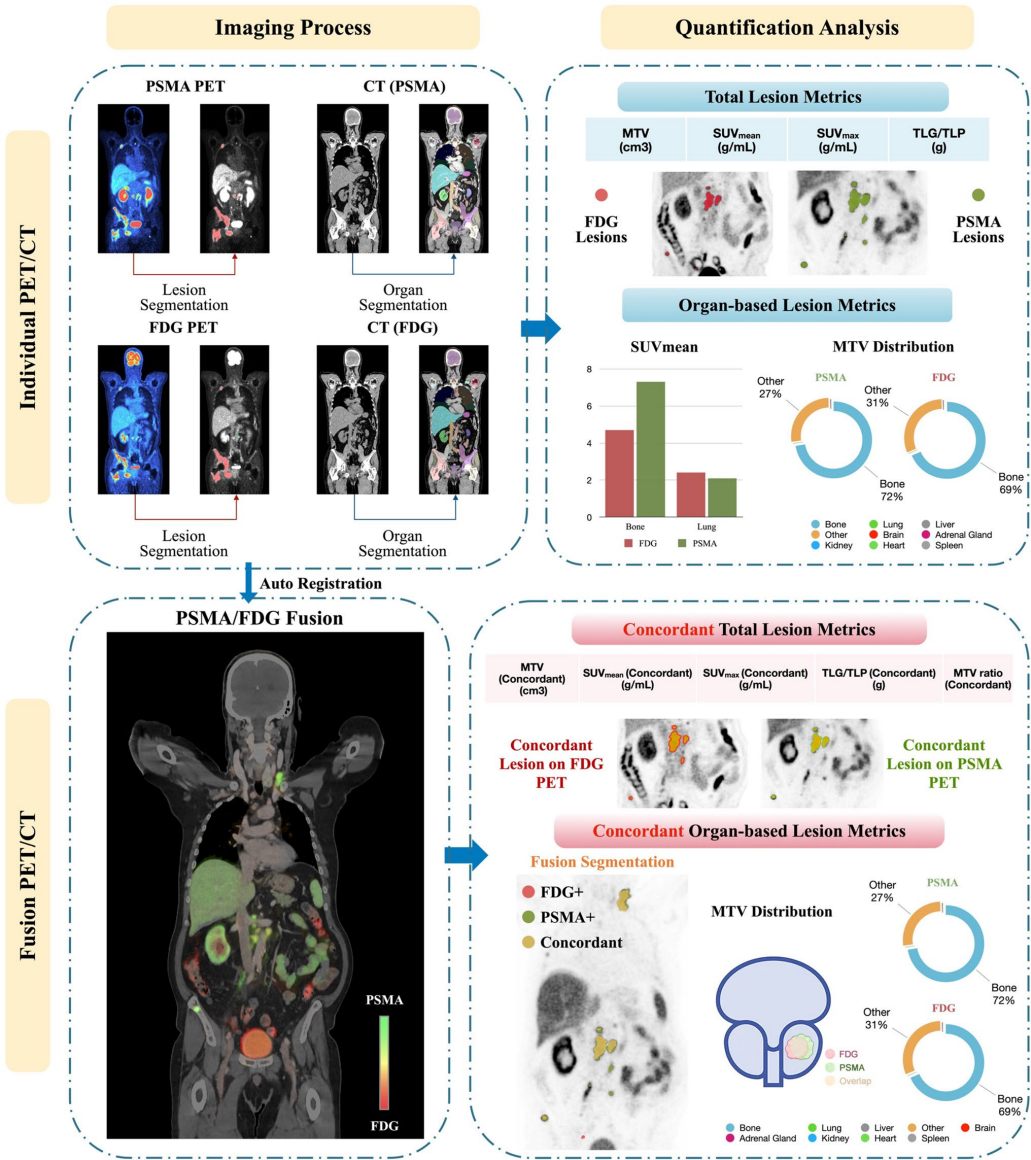
Workflow of the proposed PSMA/FDG fusion imaging analysis. The workflow consisted of independent lesion and organ segmentation on both PSMA and FDG PET/CT scans using pre-trained AI models, followed by automated registration of CT images enabled dual-tracer PET fusion. Quantitative imaging features were extracted from both individual tracer images and the fused images, incorporating total lesion metrics and organ-specific metrics to enhance predictive analysis

To enable lesion-level quantitative analysis, we developed an in-house pipeline for fusion and segmentation (Fig. 1). First, tumor lesion segmentation was performed independently on [^68^ Ga]Ga-PSMA-11 and [^18^F]F-FDG PET scans using a pre-trained deep learning model. Subsequently, rigid and deformable registration was applied to align the two CT volumes. The resulting transformation matrix was then used to register the corresponding PET images into a common anatomical space.

All segmentations and registrations were manually reviewed and refined by board-certified nuclear medicine physicians. Using the fused and co-registered PET images, concordant lesion was defined as a tumor lesion that was both identified as lesion from PSMA PET and the FDG PET. In practice, these were overlapping lesion volumes in the fused PET imaging.

For each patient, quantitative features were computed in three strata: (i) all lesions per tracer; (ii) PSMA/FDG-concordant lesions; and (iii) organ-level aggregates. Extracted features included SUV_mean_, SUV_max_, molecular tumor volume (MTV) [7], total lesion glycolysis (TLG), and total lesion PSMA (TLP) [8]. Bracket notation specifies the tracer/subset—for example, SUV_mean_[PSMA Concordant] denotes the PSMA SUV_mean_ over concordant lesions, SUV_mean_[PSMA] the SUV_mean_ across all PSMA-positive lesions, and SUV_mean_[Bone PSMA] the organ-level SUVmean across all bone metastases.

### Predictive modeling and statistical analysis

To predict patient response to PSMA-RLT, we implemented a decision tree classifier trained on imaging features from the Vienna cohort (*n* = 24). The decision tree was chosen for its interpretability and clinical relevance. Model performance was quantified as the proportion of correctly classified patients, and 95% confidence intervals were calculated using a binomial method to reflect the uncertainty [9]. To improve model robustness and reduce overfitting risk, we trained 20 decision trees with randomized initialization or sample ordering. The top five most consistently selected features across all runs were identified based on their split frequency and importance scores. From these iterations, a representative tree—containing the greatest overlap with the top-ranked features—was selected for final reporting and visualization. Model interpretation was enhanced using SHAP (Shapley Additive Explanations) to quantify the contribution of each imaging feature to the classification into PD, PR, or CR. Model performance was assessed via internal cross-validation on the Vienna cohort and externally validated using an independent cohort from the University of Augsburg.

In addition to classification modeling, we performed correlation analysis between individual imaging features and continuous PSA change (ΔPSA), using Spearman’s rank correlation. A *p*-value < 0.05 was considered statistically significant. Furthermore, organ-specific lesion features were also analyzed to explore their utility in stratifying patient groups.

## Results

### Patient population

In total, 37 patients with mCRPC who underwent dual-tracer PET/CT imaging prior to receiving PSMA-RLT were analyzed in our study. Of these, 24 patients were imaged and treated at the Medical University of Vienna, and 13 patients were recruited from the University of Augsburg. Demographic and clinical parameters of our two cohorts are demonstrated in Table 1.

**Table 1:** This is mandatory

Clinical parameters	Vienna	Augsburg
Patients—*n*	*24*	*13*
Age in years – mean (± SD)	*70.8 (8.4)*	*73.7 (5.4)*
BMI—mean (± SD)	*26.9 (4.8)*	*24.6 (3.5)*
PSA ng/mL—Mean (± SD)		
- Baseline	*187.2 (362.9)*	*849.2 (1394.6)*
- After 1. cycle	*243.8 (518.7)*	*389.7 (530.8)*
- After 2. cycles	*127.8 (322.2)*	*358.3 (490.4)*
Previous systemic treatments:		
Hormone therapy—*n* (%)	21 (87.5)	12 (92.3)
- ADT—*n* (%)	17 (70.8)	12 (92.3)
- ARTA—*n* (%)	19 (79.2)	12 (92.3)
Chemotherapy—*n* (%)	15 (62.5)	10 (76.9)
ISUP		
1—*n* (%)	1 (4.0)	0 (0.0)
2—*n* (%)	2 (8.0)	1 (7.7)
3—*n* (%)	3 (12.5)	3 (23.1)
4—*n* (%)	5 (20.8)	3 (23.1)
5—*n* (%)	13 (54.2)	6 (46.2)

*n* number of patients, *SD* standard deviation, *BMI* body mass index, *PSA* prostate specific antigen, % percentage, *RPE* radical prostatectomy, *ADT* androgen deprivation therapy, *ARTA* androgen receptor-targeted agents, *ISUP* International Society of Urological Pathology

### Patient response overview

**Fig.2:**
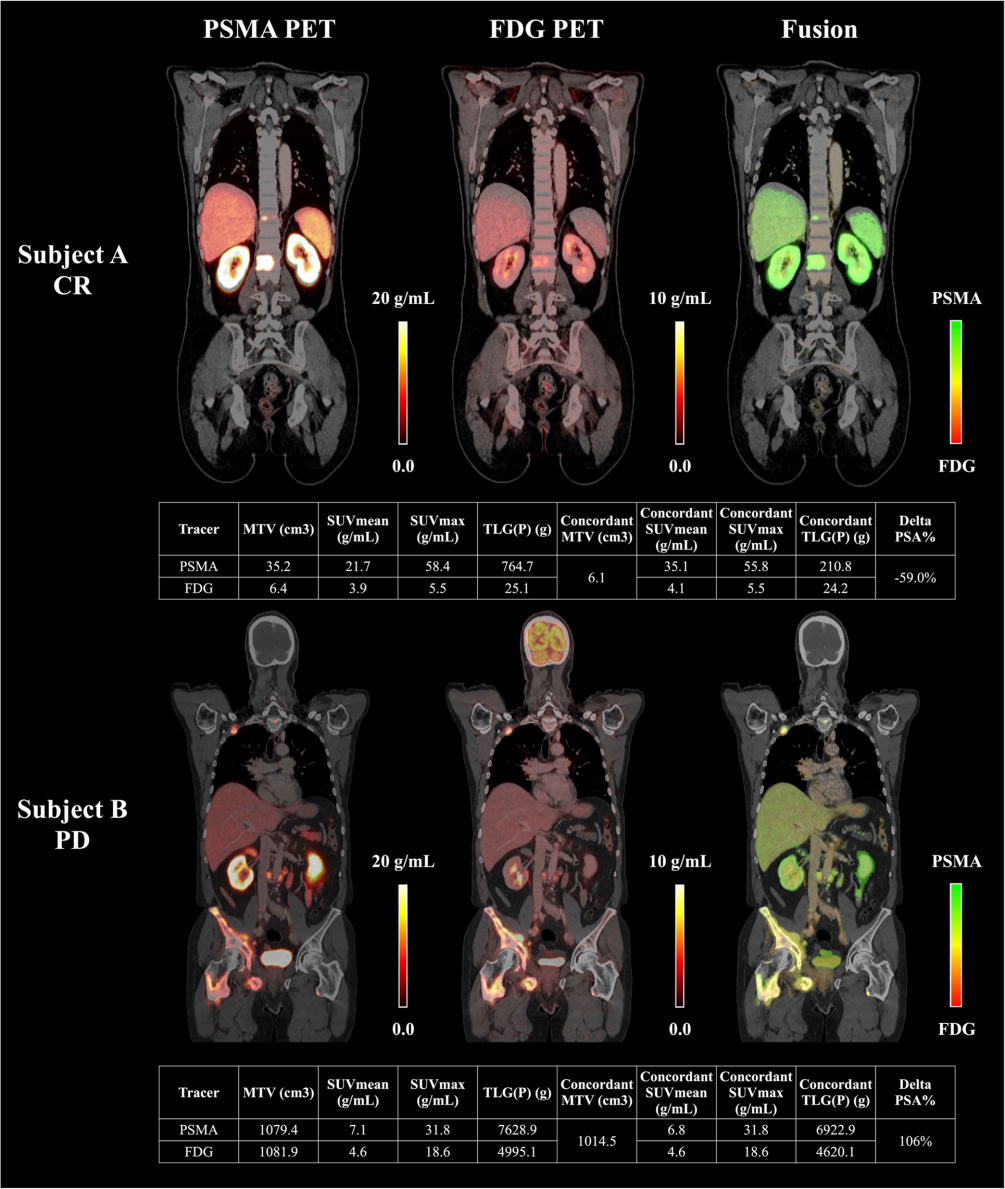
representative examples of dual-tracer pet/ct imaging and concordant lesion analysis in responders and non-responders to psma-rlt. Quantitative imaging biomarkers of concordant and non-concordant lesions, including molecular tumor volume (MTV), SUV_mean_, SUV_max_, total lesion PSMA (TLP), total lesion glycolysis (TLG), are summarized in the tables for both tracers. Coronal slices of PSMA PET, FDG PET, and fused PSMA/FDG PET (PSMA uptake = green, FDG uptake = red, Concordance = yellow) are shown for two patients: Subject A (Gleason score 10, status post androgen deprivation therapy (ADT) and chemotherapy, PSA of 9.9 µg/L at the time of imaging) exhibited metastatic involvement in bone (thoracic and lumbar vertebrae), in retroperitoneal lymph nodes, and in two intraprostatic lesions, with only low concordant tumor burden. This patient showed a complete PSA response (CR, ΔPSA = –59.0%); Subject B (Gleason score 8, status post radiotherapy to the prostate, ADT, androgen receptor pathway inhibitor and chemotherapy, PSA of 109 µg/L at the time of imaging) exhibited widespread metastases, including multiple bone lesions (upper and lower skeletal) and retroperitoneal lymph nodes, with extensive concordant tumor volume. This patient demonstrated progressive disease (PD, ΔPSA = + 106%)

Out of 24 patients in the Vienna cohort, 10 patients experienced PSA increase (PD) after two cycles of PSMA-RLT, 4 patients had minimal PSA declines (< 50% reduction, PR), and 10 patients achieved a significant PSA reduction of ≥ 50% (CR). In the Augsburg cohort (*n* = 13), 5 patients exhibited PD, 2 had PR, and 6 achieved CR following treatment. Figure 2 presents representative cases of PD and CR.

### Decision tree model performance

**Fig. 3:**
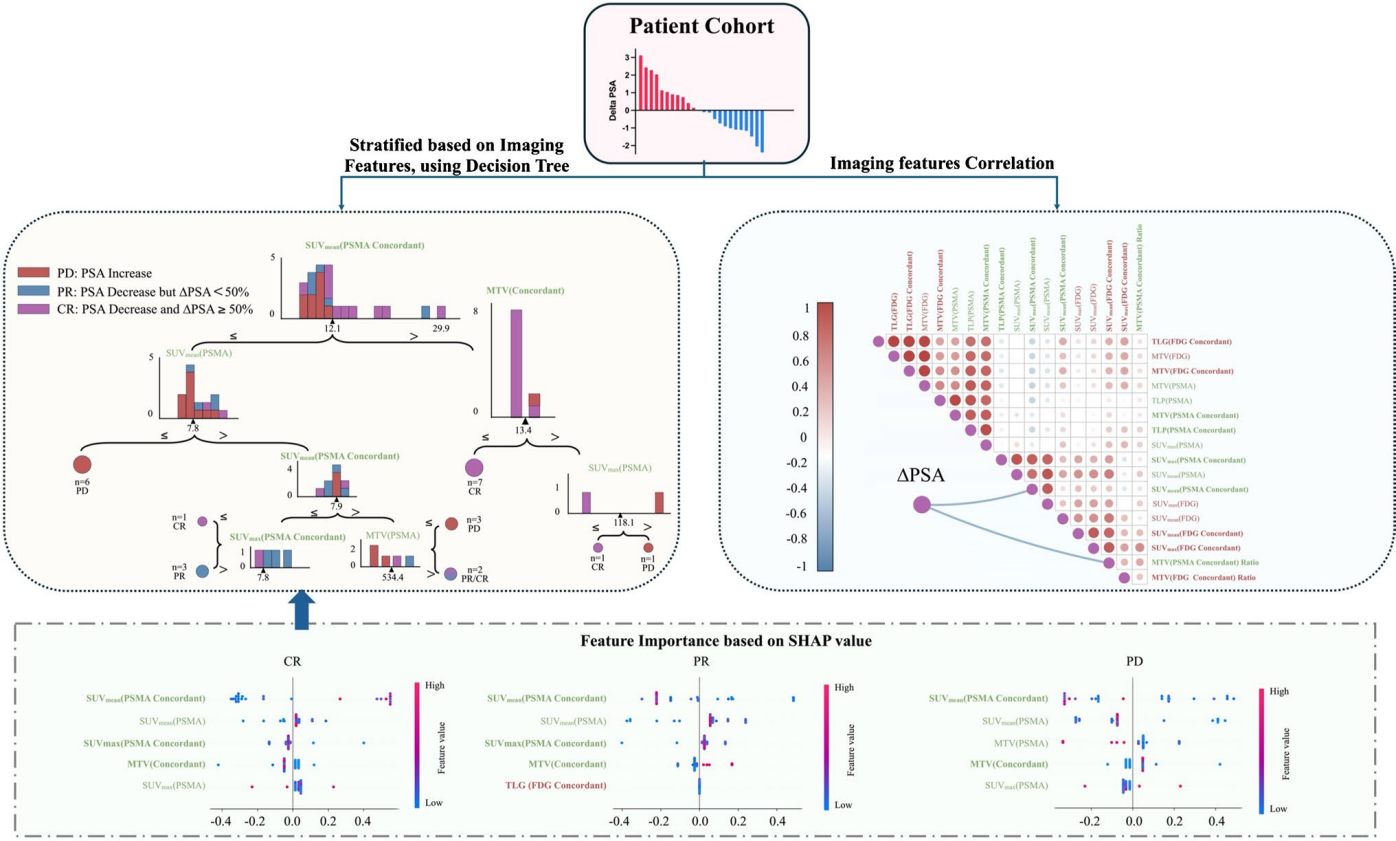
Decision tree–based stratification, feature correlation, and SHAP-derived feature importance for predicting PSMA-RLT response. Patients were categorized based on PSA response following two cycles of PSMA-RLT: progressive disease (PD, PSA increase), partial response (PR, PSA decrease < 50%), and complete response (CR, PSA decrease ≥ 50%). The decision tree classifier stratified patients into these response groups using key imaging features, with branching thresholds indicated for each node. The most important split was based on SUV_mean_[PSMA Concordant], followed by features such as SUV_mean_[PSMA] and MTV[Concordant]. Spearman correlation matrix illustrating relationships between ΔPSA and extracted imaging features. Concordant lesion metrics (SUV_mean_[PSMA Concordant] and MTV[PSMA Concordant] Ratio) showed the strongest negative correlations with PSA change. SHAP value plots demonstrating feature importance across response groups (CR, PR, and PD). SUV_mean_[PSMA Concordant] consistently ranked as the most influential feature in all categories, followed by SUV_mean_[PSMA] and MTV[Concordant]. Each dot represents a patient, with feature impact magnitude and direction shown along the x-axis

As illustrated in Fig. 3, the decision tree classifier developed on pre-therapy imaging features correctly stratified 23 of 24 patients in the Vienna cohort, yielding a classification accuracy of 95.8% (95% CI 76.9% – 99.8%). This indicates that there existed a clear separation in imaging biomarker patterns between responders (PR or CR) and non-responders (PD) in our dataset. External validation using the independent Augsburg cohort demonstrated an accuracy of 84.6% (95% CI 53.7% – 97.3%) supporting the model’s generalizability.

### Key predictive imaging features

SHAP analysis ranked the five most influential imaging features for response prediction as follows: (1) SUV_mean_ of PSMA in concordant lesions (SUV_mean_[PSMA Concordant]), (2) SUV_mean_ of all PSMA lesions (SUV_mean_[PSMA]), (3) MTV of concordant lesions (MTV[Concordant]), (4) SUV_max_ of all PSMA lesions (SUV_max_[PSMA]), and (5) MTV of all PSMA lesions (MTV[PSMA]).

**Fig. 4:**
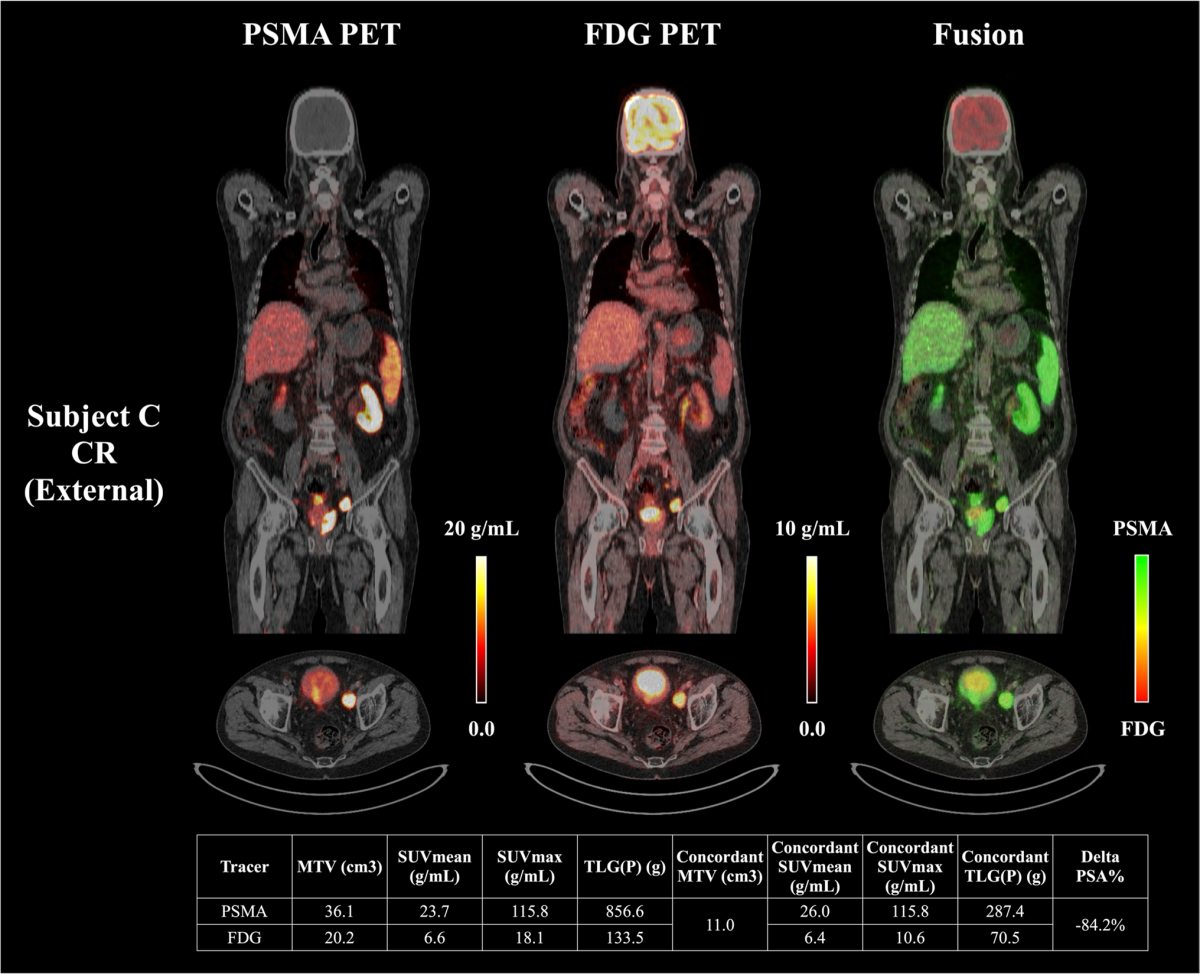
Representative case from the external Augsburg cohort demonstrating complete response, characterized by high PSMA uptake in concordant lesions. This patient exhibited complete response (CR) following PSMA-RLT, with a PSA decline of 84.2%. The SUV_mean_[PSMA Concordant] in this patient was 23.7 g/mL, exceeding the identified cutoff value (12.1 g/mL)

Among these, SUV_mean_[PSMA Concordant] emerged as the most predictive biomarker, representing the average PSMA uptake within lesions that also exhibited FDG avidity. Patients with SUV_mean_[PSMA Concordant] values exceeding 12.1 g/mL predominantly showed favorable therapeutic responses. Figure 4 illustrates a CR case from the external Augsburg cohort, characterized by a high SUV_mean_[PSMA Concordant] of 23.7 g/mL.

**Fig. 5:**
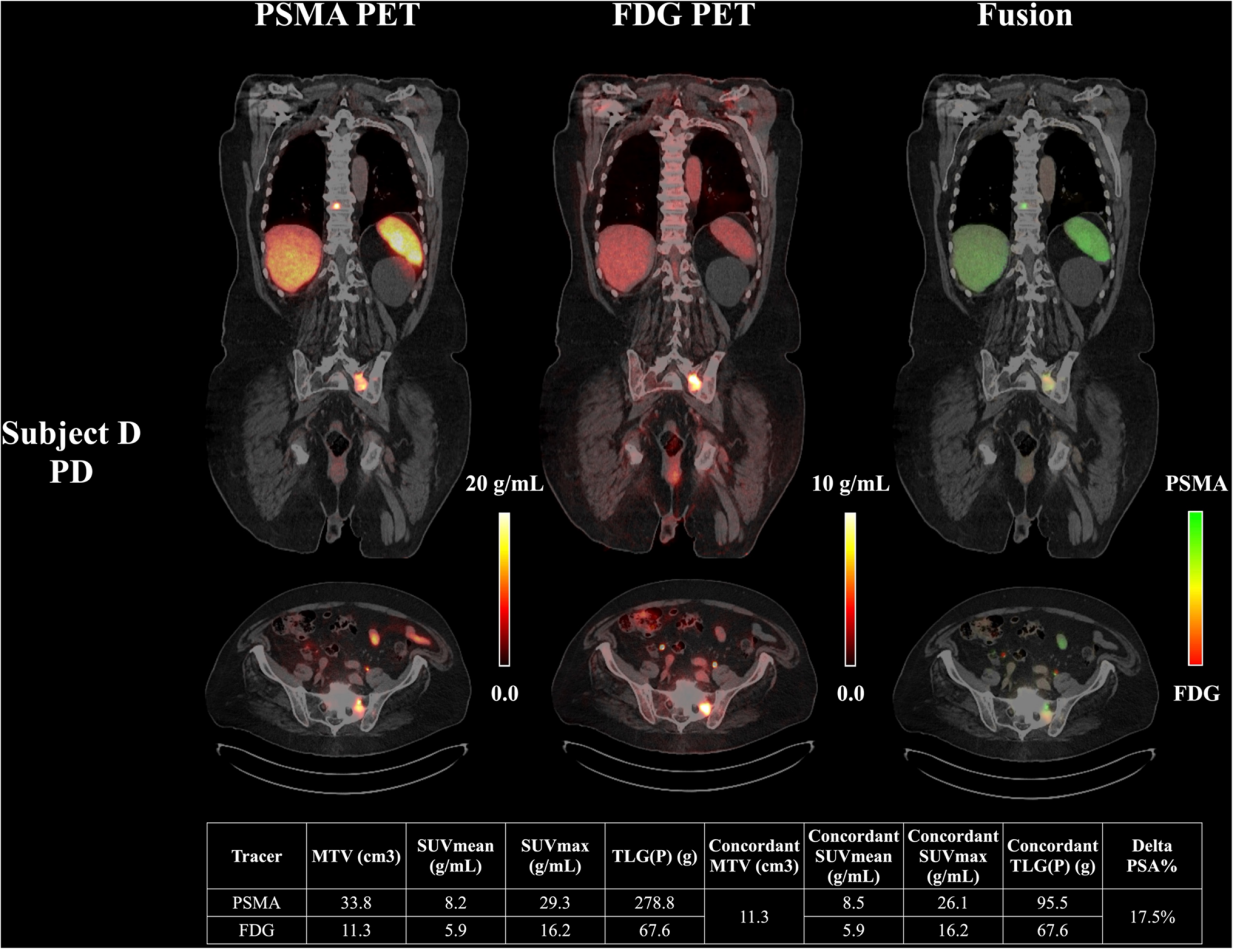
Representative case of disease progression with low PSMA uptake within concordant lesions. This patient exhibited progressive disease (PD) following PSMA-RLT, with a PSA increase of 17.5%. Although the volume of concordant lesions was relatively small (MTV[Concordant] = 11.3 cm^3^), the SUV_mean_[PSMA Concordant] was only 8.5 g/mL, falling below the predictive threshold of 12.1 g/mL identified in our cohort. This case illustrates that insufficient PSMA expression within FDG-avid lesions may contribute to suboptimal therapeutic response, even in the context of limited concordant disease burden

Conversely, lower values were associated with poor outcomes. As illustrated in Fig. 5, a representative PD case with a small concordant lesion volume (MTV[Concordant] = 11.3 mL) but SUV_mean_[PSMA Concordant] below the 12.1 g/mL threshold exhibited disease progression. Only one notable outlier deviated from this pattern: despite a high SUV_mean_[PSMA Concordant], the patient exhibited disease progression, potentially due to presence of multiple bone metastases – known and also identified in our study as a negative predictor for therapy response—with exceptionally elevated SUV_max_[PSMA] values up to 129.3 g/mL.

Whole-body PSMA metrics, including SUV_mean_[PSMA] and SUV_max_[PSMA], also contributed to outcome prediction among patients with low SUV_mean_[PSMA Concordant]. Specifically, all patients with SUV_mean_[PSMA] values below 7.8 g/mL were classified as PD, consistent with prior studies [10].

Correlation analysis further supported the importance of concordant lesion metrics. SUV_mean_[PSMA Concordant] was negatively (ρ = − 0.52) associated with PSA change (ΔPSA), with a *p*-value of 0.01. Similarly, the MTV[PSMA Concordant] Ratio—the proportion of total tumor burden that was PSMA/FDG-concordant—was also negatively correlated with ΔPSA (ρ = − 0.47, *p* = 0.02).

**Fig. 6:**
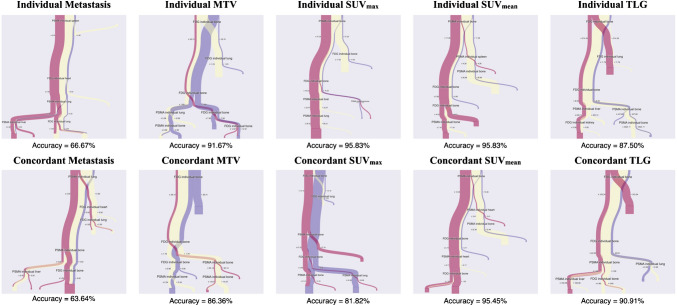
Decision tree analysis illustrating patient stratification based on organ-level imaging features. Each panel shows a decision tree constructed using a single imaging feature—MTV, SUV_max_, SUV_mean_, or TLG—to stratify patients into response categories (PD, PR, CR). The top row displays trees derived from individual tracer metrics (PSMA and FDG), while the bottom row displays trees based on metrics from concordant lesions. In all cases, features were extracted from lesions located in major organs, including the bone, liver, lung, adrenal glands, brain, kidneys, heart, and spleen

We also examined lesion-specific metrics within major organs, including bone, liver, lung, brain, adrenal glands, kidneys, heart, and spleen (Fig. 6). Organ-level SUV_max_, SUV_mean_, and Concordant SUV_mean_ achieved predictive accuracies of 95.8%, 95.5%, and 87.5%, respectively, for distinguishing among PD, PR, and CR categories. Importantly, high SUV_max_ values in bone metastases were associated with poor response, identifying them as a potential negative prognostic factor.

## Discussion

This study demonstrates that quantitative biomarkers derived from dual-tracer [^68^ Ga]Ga-PSMA-11/[^18^F]F-FDG PET/CT imaging can improve the stratification of patients with mCRPC undergoing PSMA-RLT. In particular, imaging features extracted from concordant lesions—those that are both metabolically active and PSMA-avid—were found to be strong predictors of therapeutic response. To our knowledge, this is among the first studies to apply a systematic, quantitative analysis of fused dual-tracer PET data for response prediction in this setting.

Current clinical protocols typically rely on binary eligibility criteria [11], such as requiring PSMA uptake in all known metastases and excluding patients with PSMA-negative, FDG-positive “mismatch” lesions. While useful for identifying patients unlikely to benefit, these criteria may overlook more subtle, quantitative aspects of tumor biology. In the randomised TheraP biomarker analysis, higher PSMA SUV_mean_ predicted better outcomes to [^177^Lu]Lu-PSMA-617, whereas greater FDG burden was associated with poorer prognosis—supporting quantitative dual-tracer metrics over binary rules [6]. Our results suggest that even among patients meeting conventional inclusion thresholds, variability in PSMA uptake within FDG-avid lesions—particularly concordant ones—plays a critical role in determining treatment outcomes. In this context, a substantial burden of concordant lesions with relatively low PSMA expression may represent an under-recognized risk factor for poor response.

Our findings highlight the clinical relevance of concordant lesions as key determinants of PSMA-RLT efficacy. These lesions represent tumor sites that are both PSMA-expressing (hence targetable by the therapy) and metabolically active (hence likely to drive disease progression). Consistent with this framing, Thang et al. reported short survival in men with FDG-avid disease not matched by PSMA expression [12], while Ferdinandus et al. showed opposing contributions of FDG burden (adverse) and PSMA uptake intensity (favourable) under Lu-PSMA therapy [13]. Intuitively, these are the “battleground” lesions that will determine whether PSMA-RLT succeeds or fails. Patients with high PSMA uptake in these lesions (e.g., elevated SUV_mean_) are more likely to benefit from the therapy, as the radiopharmaceutical can effectively deliver cytotoxic radiation to these targets. Conversely, if PSMA expression is insufficient, the therapy may fail to eradicate viable tumor cells, contributing to disease progression. In simple terms, patients whose aggressive tumors are “PSMA-rich” appear to derive greater benefit than those with “PSMA-poor” yet FDG-avid disease.

A central enabler of this analysis was our in-house automated image-processing pipeline, which facilitated lesion segmentation, dual-tracer image fusion, concordant lesion identification, and feature extraction at both the lesion and organ level. This approach supports standardized, scalable quantification of imaging biomarkers that go beyond conventional qualitative or visual assessment and could readily be adapted for clinical workflows. Such standardized lesion-level fusion is essential to operationalize consistently across centers.

These findings show promises to shift from binary to continuous, multi-parametric criteria for PSMA-RLT patient selection. Nonetheless, several limitations must be acknowledged. The study’s retrospective nature and modest cohort size limit its generalizability. The SUV_mean_[PSMA Concordant] threshold of 12.1 g/mL identified in this exploratory dataset should be considered hypothesis-generating. Future prospective studies with larger and more diverse populations, longitudinal follow-up, and lesion-level validation will be essential to confirm and refine these imaging biomarkers for clinical use.

## Conclusion

This two-center study suggests that quantitative metrics from dual-tracer [^68^ Ga]Ga-PSMA-11/[^18^F]F-FDG PET/CT—particularly features of PSMA/FDG-concordant lesions—may improve prediction of response to PSMA-RLT in mCRPC. These candidate biomarkers could support more personalized treatment decisions and, with prospective validation and protocol harmonization, may complement or refine current binary eligibility criteria to enhance patient selection.

## Data Availability

Data are available for legitimate researchers who request it from the corresponding author.
